# The impact of stillbirth on bereaved parents: A qualitative study

**DOI:** 10.1371/journal.pone.0191635

**Published:** 2018-01-24

**Authors:** Daniel Nuzum, Sarah Meaney, Keelin O’Donoghue

**Affiliations:** 1 Department of Obstetrics and Gynaecology, University College Cork, Cork University Maternity Hospital, Wilton, Cork, Ireland; 2 National Perinatal Epidemiology Centre, University College Cork, Cork University Maternity Hospital, Wilton, Cork, Ireland; 3 Irish Centre for Fetal and Neonatal Translational Research (INFANT), Department of Obstetrics and Gynaecology, University College Cork, Cork, Ireland; UniSA, AUSTRALIA

## Abstract

**Purpose:**

To explore the lived experiences and personal impact of stillbirth on bereaved parents.

**Methods:**

Semi-structured in-depth interviews analysed by Interpretative Phenomenological Analysis (IPA) on a purposive sample of parents of twelve babies born following fetal death at a tertiary university maternity hospital in Ireland with a birth rate of c8,500 per annum and a stillbirth rate of 4.6/1000.

**Results:**

Stillbirth had a profound and enduring impact on bereaved parents. Four superordinate themes relating to the human impact of stillbirth emerged from the data: maintaining hope, importance of the personhood of the baby, protective care and relationships (personal and professional). Bereaved parents recalled in vivid detail their experiences of care following diagnosis of stillbirth and their subsequent care. The time between diagnosis of a life-limiting anomaly or stillbirth and delivery is highlighted as important for parents as they find meaning in their loss.

**Conclusions:**

The impact of stillbirth on bereaved parents is immense and how parents are cared for is recalled in precise detail as they revisit their experience. Building on existing literature, these data bring to light the depth of personal experience and impact of stillbirth for parents and provides medical professionals with valuable insights to inform their care of bereaved parents and the importance of clear and sensitive communication.

## Introduction

Stillbirth is without question one of the most distressing experiences of bereavement with long-lasting impact for bereaved parents, healthcare professionals and society at large.[1–4] The diagnosis that a baby will not survive or has already died *in utero* brings with it a bewildering array of emotional distress where birth and death collide and parents move from a trajectory of expectation to one of grief.[5–7] How parents are cared for during this time can have long-lasting consequences, both positive and negative.[7, 8]

The psychosocial impact of stillbirth on parents is well documented in the published literature and a renewed global focus in 2016 on the prevalence of stillbirth and its associated impact has sought to heighten public awareness of stillbirth as a societal issue.[1, 9–11] Stillbirth in Ireland (where this study was conducted) is defined as ‘‘a child born weighing 500 grammes or more or having a gestational age of 24 weeks or more who shows no sign of life’.[12] The incidence of stillbirth in Ireland is 1 in 238 births.[13] The lived experiences of bereaved parents contribute an invaluable insight into the profoundly human experience of this particular grief in obstetrics.[6, 7, 14–19] Recently published metasyntheses and a systematic review have affirmed the importance of parental experience following stillbirth and while most studies have explored the impact of stillbirth by way of online questionnaires, fewer have used face to face interviews with bereaved parents.[1, 20, 21]

The objective of this study was to qualitatively explore through interview and analysis the personal impact of stillbirth on bereaved parents and to research the lived experience of parents who received a diagnosis that their baby had died or would die before birth. This approach brings to a deeper level the understanding of stillbirth and how it impacts on parents.

## Methods

Qualitative methods are used to understand complex social processes, to capture essential aspects of a phenomenon from the perspective of study participants, and to uncover beliefs, values, and motivations towards care and service provision. Interpretative phenomenological analysis (IPA) is a well-established qualitative research methodology that has grown from the field of health psychology and is used increasingly in health science research to understand how people make sense of their experiences.[3, 22, 23]

### Sample

**Fig 1:**
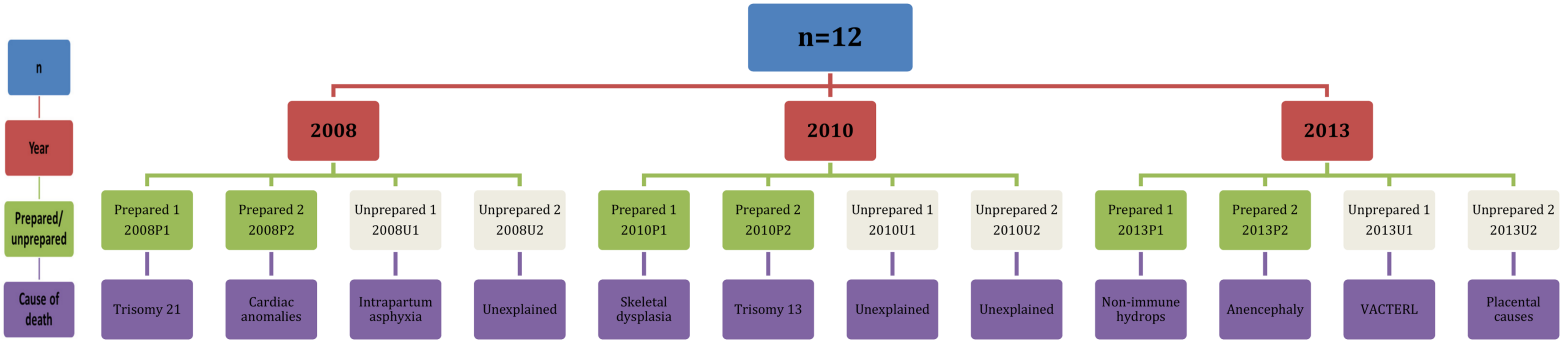
Details of study participants and cause of death.

Twelve mothers and five fathers participated in the study. Details of participants including year of stillbirth, whether parents were prepared or unprepared for the death of their baby and the cause of death are illustrated at Fig 1. The parents of 50% of the babies in the sample from each year had received a diagnosis *in utero* that their baby had a life-limiting condition and was unlikely to survive and therefore had time to prepare for the anticipated death of their baby. The remaining 50% of parents had experienced an unanticipated stillbirth.

### Recruitment

Following ethical approval from the Clinical Research Ethics Committee of the Cork Teaching Hospitals (Ref. No: ECM 4 (pp) 06/03/12) a purposive sample of bereaved parents of twelve babies who had died following stillbirth at an Irish tertiary maternity hospital (four babies from three individual years from when the hospital opened in 2008) were invited to participate in the study. Inclusion criteria were that the participants had been cared for at the study hospital, were not currently pregnant, were over eighteen years old and had not previously indicated that they did not wish to be contacted by the hospital for study purposes.

Bereaved parents are a vulnerable population, and as the primary relationship was between the hospital and mothers, the initial contact in the study was made by a bereavement and loss midwife specialist known to bereaved mothers to ascertain if they would be willing to receive an invitation to participate. All those contacted were willing to participate in the study. Each bereaved mother then received a personal invitation to participate in a semi-structured interview with the researcher, with the stated aim to explore the spiritual and pastoral needs of bereaved parents following stillbirth and what their experiences of care were. Each participating mother was invited to extend the invitation to her partner to participate in the study. The spiritual and pastoral dimensions of the study are published elsewhere.[24, 25]

### Data collection

A semi-structured interview topic guide with open questions was developed by the authors based on their experience working in a perinatal bereavement specialist team. A copy of the interview schedule is included at S1 Appendix. Semi-structured interviews were conducted to ensure a consistency of topics covered and also to allow for the lived experiences of bereaved parents to be captured. This ideographic approach invites the sharing of important insights from the world of the participant and facilitates the emergence of topics of importance to the participant that might not have been thought of by the researcher.[23]

Following written consent each interview took place in a private environment without interruption at a location and time of the participants’ choosing. Most participants (n = 14) were interviewed in their home environment and the remaining (n = 3) chose to return to the study hospital. Interviews lasted between 31 and 104 minutes, were digitally recorded and subsequently transcribed verbatim. Transcripts were anonymised to protect the identity of the participants. Following transcription and before analysis, each transcript was checked for accuracy against the original recordings by the researcher.

### Analysis

The data were analysed using IPA. Data analysis is thorough and undertaken in five steps: (i) Familiarisation of the transcripts–*listening to recordings*, *reading transcripts*, *reviewing notes of initial impressions*; (ii) Preliminary themes identified–*this is done on a case by case basis; it involves focussing on key words and phrases that were coded; (iii)* Themes are grouped together as clusters; *related themes are arranged together*; (iv) The creation of a master table of themes; *which themes have commonalities or contradictions*? *These are then developed into superordinate themes which are made up of subordinate themes*; (v) The integration of cases; *this is where one moves from the individual to the whole sample; moving from one transcript to the next and compare and contrast the themes—is there a pattern emerging from the sample as a whole*? [23]

The data were analysed by two members of the research team separately. Consensus was formed on the emergence of superordinate and subordinate themes with the senior author. Data were managed using NVIVO Version 10 (QSR International).

## Results

**Fig 2:**
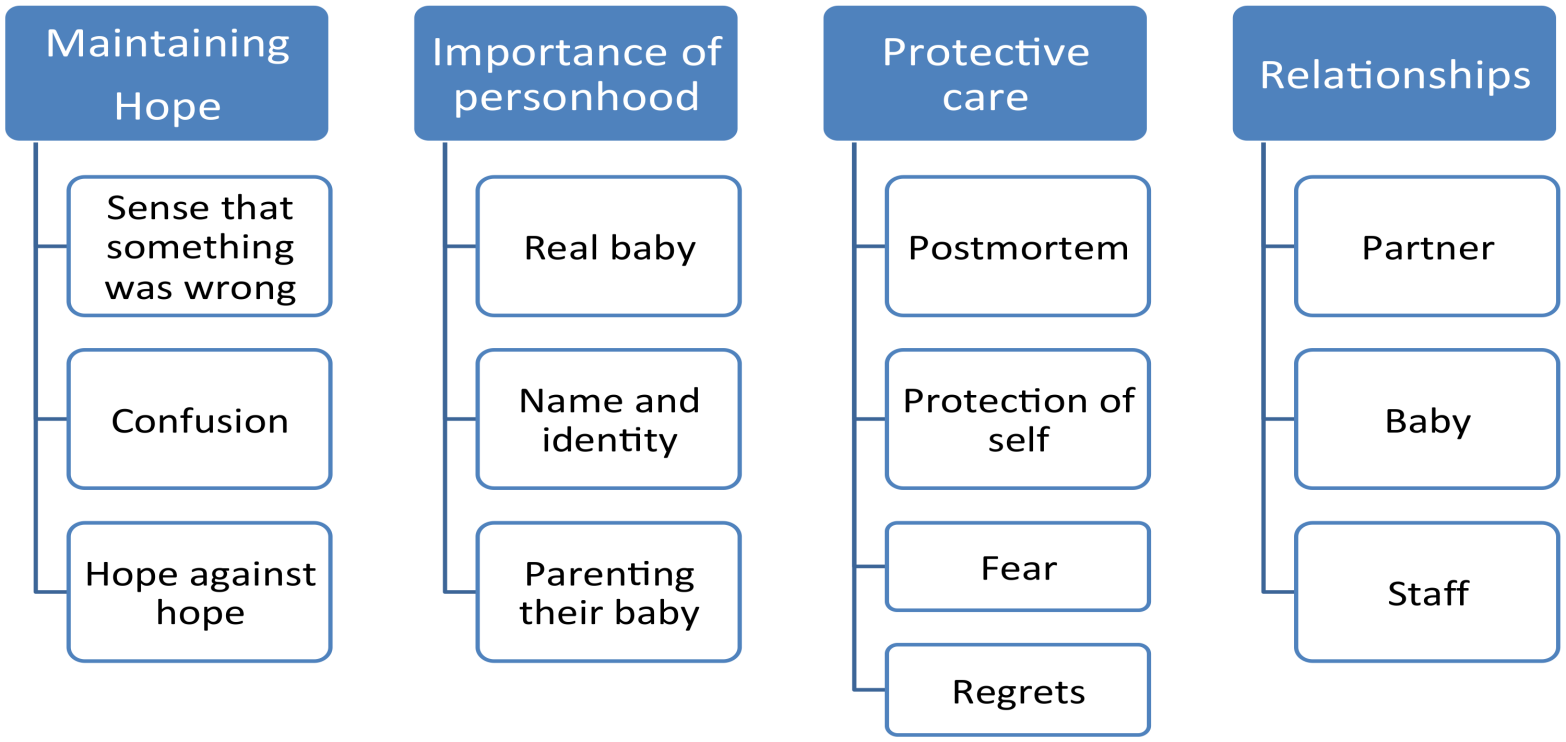
Superordinate and subordinate themes.

Following analysis of the data, four superordinate themes emerged related to the impact of stillbirth on parents: maintaining hope, importance of personhood, protective care and relationships. A figure of superordinate themes and associate subordinate themes is illustrated in Fig 2.

Direct quotes are used in the paper to demonstrate the results and to highlight each theme in the study. Each quotation is referenced by year of bereavement and whether a baby had an antenatal diagnosis of a life-limiting condition with an expected outcome of stillbirth (indicated by a P) or whether their stillbirth was unanticipated (indicated by a U).

### Theme 1: Maintaining hope

All parents spoke about how it was important for them to maintain hope even in the midst of devastating sadness and loss. The superordinate theme of hope was expressed both in terms of how hope was an important part of coping with loss, and also a struggle to find hope when everything seemed hopeless. This theme evolved from hopelessness at diagnosis to maintaining hope during the remainder of a pregnancy and into the future. The subordinate themes to emerge were: sense that something was wrong, confusion and hope against hope.

#### (i) Sense that something was wrong

The subordinate theme of ‘sense that something was wrong’ ranged from a feeling of premonition by mothers that something might be wrong with their baby or pregnancy before receiving a diagnosis, to an attachment of significance to particular events or experiences retrospectively when parents revisited their experiences afterwards. For those who had an unexpected stillbirth the sense that something was wrong was associated with panic and fear. One mother when she felt that something was wrong recalled:

*“I remember lying up on my bed hitting my tummy trying to get some response…I went to the GP and I literally burst in the door and collapsed to the floor where I was hysterical*.*”* ^2008U1^

#### (ii) Confusion

Parents expressed the inner conflict they experienced when they heard the news that their baby had died. This was mostly expressed by those who received an unexpected diagnosis that their baby had died during an otherwise healthy pregnancy. The sudden shift in emotion from expectancy to devastation followed by the finality of death created inner emotional and stress-related conflict. For two mothers this was experienced as an out-of-body experience where they felt detached from what was happening.

*“I was just sitting there looking at him* (baby); *I didn’t feel anything for ages …* (crying and barely able to speak during this part of interview). *It was like I couldn’t believe he was dead*.*”* ^2013P2^

When parents expressed confusion it was part of their hope that the news received was untrue. For some parents their confusion stemmed from lack of clarity from staff especially at the time of diagnosis. One parent described this confusion:

*“It was one of the toughest nights … I didn’t expect anything to be wrong* (long pauses, crying and deep breaths) *It was the longest time I had to wait for anything and he* [doctor] *just did the scan and said there was fluid in the baby’s stomach and he didn’t really explain what was wrong … I was just in a daze walking out the door*.” ^2013P2^

#### (iii) Hope against hope

Many parents spoke of a sense of trying to maintain ‘hope against hope’ that the diagnosis about their baby was wrong and that their baby might survive. For parents who had an unexplained stillbirth they tried to maintain hope in the midst of panic, fear and confusion from the moment they suspected something was wrong until they received confirmation of the reality of their baby’s death at the time of the baby’s birth.

*“He* [husband] *said ‘there’s no heartbeat’ and I said ‘we’ll wait*, *we’ll see’*. *I still continued in labour as if my baby could still be alive*. *I said*, *‘I’m not going to accept there’s no heartbeat until I see my baby’*. *And then the baby came and he wasn’t alive so I had no words*, *just no words*.*”* ^2010U2^

The subordinate theme of hope against hope was characterised by the desire of some parents to do something that might change the outcome for their baby. For some this meant hoping that the obstetrician had got the diagnosis wrong, and for others who received a diagnosis in pregnancy, it was changing their lifestyle habits such as eating more healthily, exercising or taking bedrest.

### Theme 2: Importance of personhood

The importance of the personhood of their baby was a dominant superordinate theme for all parents. Parents spoke about the uniqueness of their baby and how each baby had an enduring importance as a human being that mattered.

*“He played a big part in changing our lives in a year*. *Not only in his own presence … he also impacted in other areas of our lives as well*. *He was a very powerful little person*.*”* ^2013U2^

Important subordinate themes in the superordinate theme of personhood were recognition of a stillborn baby as a real baby, the baby’s unique identity, and how parents actively parented their baby as they would a live baby.

#### (i) Real baby

The importance of a stillborn baby being recognised and treated in the same way as every other baby was expressed by most parents. Parents who had other children expressed that they felt the same love and care towards this baby as they had towards their other children.

*“He was perfect*, *he was exactly the same as* [every other baby] *in one sense it was nice but it wasn’t nice*.*”* ^2013U1^

#### (ii) Name and identity

Parents created an identity for their baby both antenatally and also in how they related to their baby following their birth. Identity was an important way of relating when they were no longer physically present. Central to the identity was the baby’s name -all parents gave a name to their baby. The identity of the baby as being part of the family was important, whether parents had other living children or not. Even when parents had no living children they still described their baby’s place as being part of their family.

*“We brought him home*. *He was in all their houses*, *my mum and dad*, *they all had cuddles*, *my nieces and nephews all had photos*. *… He is the most thought-of baby; he’s not forgotten by anyone*. *He’s always remembered*.*”* ^2008U1^

#### (iii) Parenting their baby

A subordinate theme for all parents was the importance of opportunities they had to parent their baby.

Many parents emphasised the value of the finite time between the birth of their baby and their burial or cremation.

*“I wanted to take him home*. *I wanted as much time with him as we could … I suppose we knew we wouldn’t have long with him before we buried him*.*”* ^2008U1^

### Theme 3: Protective care

Each parent displayed a strong protective instinct towards their baby. The desire to protect their baby was also a challenge for parents as they faced the reality of their own powerlessness and inability to protect their baby from inevitable death following a life-limiting diagnosis. One father spoke about his sense of personal pain and how he would have gladly stepped into the place of death himself if that would have saved his son’s life.

*“We are just suffering our way through it … you just have to take one hour at a time … I wish I could have gone and he could have stayed … and let him have a life* (long silence).*”* ^2013P2F^

The following subordinate themes in the data were part of the overall superordinate theme of protective care: post-mortem examination, protection of self, fear and regrets.

#### (i) Post-mortem examination

The issue of post-mortem examination was raised by a number of parents as part of their sense of protective care for their baby where they wished to protect their baby from any further tests and interventions. Two parents expressed that they did not want their baby to undergo a post-mortem examination as it felt like an unnecessary ‘extra ordeal’ for their baby. In both cases these were unanticipated stillbirths.

For two parents the reality of post-mortem was a distressing experience for them after they had gone home. In one case, although they had signed a formal post-mortem consent form, the couple had not understood that their baby’s organs would be retained and then subsequently returned to them. This couple spoke very vividly of the distress caused to them when they received a telephone call ‘out of the blue’ from the hospital to collect their baby’s organs. They found it to be a traumatic experience to return to the hospital to collect their baby’s organs to bring home for burial. This couple buried their son’s organs in darkness in his grave late at night.

*“Then after I don’t know how long*, *a month or six weeks the phone rang one day and said that baby’s organs were back* [voice breaking and finding it hard to continue speaking] *It was like going back to rock-bottom again*.*”* ^*(2013U2)*^

One mother spoke in a very positive way of the transforming experience when she received her baby son back from his post-mortem examination which she was initially reluctant to give her consent for.

*“The most wonderful thing happened when our baby came back* [from post-mortem]. *I smiled*, *I was full of joy*. *I saw my baby in a baby-gro of blue and white and all of a sudden things changed*. *I can’t explain it*. *I said ‘wow*, *look at our baby’*. *From then on he became someone to me*.*”* ^2010U1^

#### (ii) Protection of self

When parents received a diagnosis that their baby might not survive, theyspoke of not wanting to share the news publicly and the need to protect themselves from ordinary social interactions when they might be placed in a situation of having to explain that something was wrong.

*“I didn’t say anything to anyone in work*, *just close family*, *what was happening and let-on to the outside world that it was just a pregnancy as normal*. *I didn’t want people to keep asking me what was the story*?*”* ^2010P2^

Some bereaved parents felt a sense of exposure when they met other parents and their babies as they were leaving hospital or other pregnant women when waiting to have their stillbirth confirmed. This experience was evident in the data from 2008 but did not appear in the data from later years.

*“When I look back I found that hard … the room … is it like a triage room*? *I could hear other women with their heartbeats obviously … I remember lying there hearing the other women and one woman was in labour and it upset me*.*”* ^2008U1^

Encountering other parents and babies evoked jealous feelings and painful reminders of the reality of their loss. Parents expressed how they appreciated being cared for in a single room in a dedicated part of the hospital.

#### (iii) Fear

Fear was a common thread in the protection of self, ranging from the unknown of what happened and how to cope with the impending birth and death of their baby. Parents expressed fear about what their baby might look like and how others would react.

“I remember thinking that she might feel cold and I’d be afraid.” ^2010P1^

#### (iv) Regrets

The subordinate theme of regrets was identified as parents in hindsight had regrets about aspects of their care or decisions they had made. For some parents it was linked with not parenting their baby and opportunities they did not have or did not avail of.

*“I know it might sound ridiculous but I’d like to have seen all of him*.*”* ^2010P2^

Others had regrets that they did not respond immediately to a symptom which in hindsight they felt was significant.

*“It was really weird that day* .. *about half six that morning*, *I had such movement that just woke me*. *When I look back I don’t know if that was when it happened … I don’t know*.*”*^2008U1^

These regrets were expressed as part of a revisiting of their story in trying to understand why their baby had died.

### Theme 4: Relationships

All parents expressed that the stillbirth of their baby had impacted on relationships; some positively but most negatively. The data revealed three different patterns of relationships: with partners, with their baby, and with staff.

#### (i) Relationship with partner

All but three participants reported that the death of their baby had impacted negatively on their relationship with their partner. Most parents said that they found it hard to communicate with their partner about their feelings of grief.

*“In the beginning we talked and cried but we don’t talk about it much anymore because it’s too painful*. *It just breaks your heart … everything we have done together has been ruined*, *tainted*.” ^2013P2^

In contrast, one mother was very expressive about how her relationship with her partner was strengthened following their baby’s diagnosis of a life-limiting condition.

*“Like me and my husband*, *we were never so united*. *We spent a lot of time together*, *we talked about everything*.*”* ^2010P1^

#### (ii) Relationship with baby

All parents said that they felt a strong relationship with their baby during pregnancy and how everything seemed to be ‘normal’ until they received their diagnosis of a life-limiting condition or stillbirth.

The diagnosis of a life-limiting condition allowed parents time to prepare for the impending death of their baby. Parents appreciated the time they had between diagnosis and death/birth to create memories with their baby before their baby’s death and how this time helped them in their grieving process. Most parents expressed that they valued the support of the multidisciplinary team during this period.

*“I was just going to be grateful for what I could get and for every kick … Getting the diagnosis early was a blessing because I was able to enjoy everything*.*”* ^2013P3^

Most parents expressed that they had a strong ongoing relationship with their baby. This was expressed by a sense of ‘closeness’ and proximity to the spirit of their baby. Fathers expressed that they only started to bond with their baby following their birth. This was expressed by some fathers as a source of personal tension as they were envious of the relationship their partner had with their baby.

*“I think* [partner] *had a lot closer connection to him than I had*, *because I suppose I see my time with him as*, *when he was born to when he was buried*. *… I remember thinking he’s my son but he’s not* (very upset).*”* ^2010P2F^

Four fathers expressed that they had a more private ongoing relationship with their baby. One father shared how he felt very close to his son when he visited his grave which he did every night.

*“*[I feel close to him in the graveyard] *I just prefer it if there was no one else in the graveyard*. *I would definitely only feel it when I’m there on my own with him*.*”* ^2010P2F^

#### (iii) Relationship with staff

All parents spoke of the relationships they had with the staff who cared for them during their pregnancy and following the birth of their baby. The data revealed that how staff interacted with parents left a lasting impression that was equally vivid across the three year groups. In particular, how parents experienced communication and care at key moments such as diagnosis or during fetal scanning were recalled in precise detail. The experiences that parents shared were examples of what is considered both good and bad practice in the care of bereaved parents.

*“I could feel the kindness off her* [consultant]. *I knew she really cared*.*”* ^2013P2^

Parents who had negative experiences recalled them with anger towards the staff involved.

*“During the first scan she was measuring this and measuring that and she told me she was a trainee*, *and in my own head I was going ‘go out and get someone who knows what they are doing ‘… she said ‘maybe I’m doing something wrong*, *go away and come back in two weeks*.*”* ^2010P2^

## Discussion

### Main findings

The impact and burden of stillbirth is immense for bereaved parents having an ongoing influence on many aspects of their lives and relationships. Bereaved parents recalled in precise detail the events and experiences leading up to, surrounding and following the diagnosis of a life-limiting condition or stillbirth of their baby. Consonant with recently published meta-syntheses, the experiences of parents in this study contribute valuable personal insights into the depth of perinatal grief.[20, 21, 26, 27]

In an Irish context where termination of pregnancy is not a permissible option this study sheds important light on the importance of a perinatal palliative care approach for babies and their parents following a diagnosis of a life-limiting condition *in-utero*.[28] In keeping with a study by O’Connell *et al* the findings in this study highlight that for parents who receive a life-limiting diagnosis, that is likely to result in the death of their baby, the time between diagnosis and death/birth is valuable time where they can be helped to process their loss and find meaning with the support of a multidisciplinary perinatal bereavement team.[29] Parents who experience an unexpected stillbirth do not have time to prepare; however the time following diagnosis and the immediate care before, during and after birth are valuable opportunities for sensitive bereavement care. Conversely, as highlighted in the theme ‘hope against hope’ the time following a diagnosis of a life-limiting condition also reveals the reality of emotional conflict when parents continue to hope that a diagnosis might be wrong.

The challenging conversations concerning post mortem consent and associated procedures were raised by the participants in this study. The data revealed the impact of confusion concerning post mortem consent and practice and the strength of parental protectiveness. Due to falling perinatal post mortem rates this is an area that deserves renewed attention. This study supports a recently published study concerning the protective stance taken by bereaved parents towards their baby and how parents understand the role of post mortem examination.[30] These findings are a timely reminder of the importance of sensitive and unambiguous consenting procedures at what is an inevitably emotional time for parents.[30–34]

The ‘sense that something was wrong’ that emerged for some mothers in this study is an important insight in light of increasing awareness of risk factors for stillbirth.[35] In addition to the well documented modifiable risk factors, the maternal ‘gut instinct’ that something might be wrong, as identified by Warland *et al*, also featured in our study.[36] The data from this study demonstrated that parents recalled their experiences as they sought to make sense of what had happened. Consonant with recent studies these data support the introduction of public health initiatives to raise awareness of potentially modifiable risk factors for stillbirth.[35, 36]

The impact of stillbirth on relationships is highlighted in this study. The data identified how the experience of the diagnosis of a life-limiting condition or stillbirth impacted both positively and negatively on relationships between parents, with their baby and with healthcare professionals.[30, 37] Of note, parents did not refer to other relationships with family or friends other than being cautious about sharing their diagnosis socially. This might suggest that bereaved parents experience a sense of isolation from wider supportive relationships and is worthy of further study.

How parents experience care at a traumatic time is influenced by the relationship, attitudes and behaviour of staff and the environment of care. Building on a previous study by Downe *et al*, (2013) the findings from this study concerning the relationship between parents and staff highlight the importance of good communication and empathic care and how this impacts on the overall experience at a distressing time.[37] As the participating parents remembered in detail their experiences from staff at key moments of care such as diagnosis and during scans, the importance of good communication and sensitive care is highlighted. Clinicians can not change the inevitable and devastating news that a baby has died but they can change how this news is communicated and the care they can give to parents.

### Strengths and limitations

The strengths of this study are that it focuses in an in-depth way on the impact of stillbirth for bereaved parents using qualitative methodology to reveal the lived experiences and associated meanings attributed to stillbirth. As this was a qualitative study the results pertain to parents and healthcare professionals from one maternity hospital. These data are particular to the participants and are not generalisable to a global population, however the insights and experiences are likely to have transferrable commonalities for other bereaved parents. In addition, these findings have importance beyond the sample studied and contribute valuable insight for clinicians caring for parents following stillbirth and for wider maternity service development.

A limitation of the study is that fathers were recruited through their partners. This may have impacted on the lower level of participation by bereaved fathers.

## Conclusion

How parents are cared for following the diagnosis of a life-limiting condition for their baby in pregnancy or following stillbirth has considerable impact on their grieving process. The importance of a clear, supportive and sensitive post mortem consent procedure is highlighted as is the importance of appropriately trained staff and professionally integrated bereavement care.

Complementing the renewed call to reduce the global burden of stillbirth this study keeps a focus on the intensely personal and human experience of stillbirth. This study adds valuable new qualitative data from the experiences of bereaved parents following face-to-face interviews to build on other studies that have been from a mainly quantitative perspective or using online data collection. This also provides important insights concerning communication, sensitive care and postmortem consent for medical practitioners who provide care for parents following the diagnosis of a life-limiting anomaly or stillbirth. For every stillborn baby, in addition to the loss of that baby in society, there are grieving parents who carry this loss for the rest of their lives. Maternity healthcare professionals caring for parents when their baby has died can learn valuable lessons from the voices of bereaved parents. It is hoped that this continues to improve the overall care provided at a time of distress for both clinicians and parents.

## Supporting information

S1 Appendix Interview schedule. (DOC)

## References

[1] Heazell AE, Siassakos D, Blencowe H, Burden C, Bhutta ZA, Cacciatore J, et al Stillbirths: economic and psychosocial consequences. Lancet. 2016;387(10018):604–16. doi: 10.1016/S0140-6736(15)00836-3 . 26794073 10.1016/S0140-6736(15)00836-3

[2] Flenady V, Boyle F, Koopmans L, Wilson T, Stones W, Cacciatore J. Meeting the needs of parents after a stillbirth or neonatal death. BJOG: an international journal of obstetrics and gynaecology. 2014;121 Suppl 4:137–40. doi: 10.1111/1471-0528.13009 . 25236648 10.1111/1471-0528.13009

[3] Nuzum D, Meaney S, O’Donoghue K. The provision of spiritual and pastoral care following stillbirth in Ireland: a mixed methods study. BMJ Support Palliat Care. 2016;6(2):194–200. doi: 10.1136/bmjspcare-2013-000533 . 24916197 10.1136/bmjspcare-2013-000533

[4] Nuzum D, Meaney S, O’Donoghue K. The impact of stillbirth on consultant obstetrician gynaecologists: a qualitative study. BJOG: an international journal of obstetrics and gynaecology. 2014;121(8):1020–8. doi: 10.1111/1471-0528.12695 . 24589177 10.1111/1471-0528.12695

[5] Badenhorst W, Hughes P. Psychological aspects of perinatal loss. Best Practice & Research Clinical Obstetrics & Gynaecology. 2007;21(2):249–59. 17196434 10.1016/j.bpobgyn.2006.11.004

[6] Erlandsson K, Saflund K, Wredling R, Rådestad I. Support After Stillbirth and Its Effect on Parental Grief Over Time. Journal of Social Work in End-of-Life & Palliative Care. 2011;7(2/3):139–52. doi: 10.1080/15524256.2011.593152 21895434 10.1080/15524256.2011.593152

[7] Cacciatore J, Bushfield S. Stillbirth: The mother’s experience and implications for improving care. Journal of Social Work in End-of-Life & Palliative Care. 2007;3(3):59–79. 2007-19985-004. 10.1300/J457v03n03_06 18077296

[8] O’Connell O, Meaney S, O’Donoghue K. Caring for parents at the time of stillbirth: How can we do better? Women Birth. 2016;29(4):345–9. doi: 10.1016/j.wombi.2016.01.003 . 26916147 10.1016/j.wombi.2016.01.003

[9] Flenady V, Wojcieszek AM, Middleton P, Ellwood D, Erwich JJ, Coory M, et al Stillbirths: recall to action in high-income countries. Lancet. 2016;387(10019):691–702. doi: 10.1016/S0140-6736(15)01020-X . 26794070 10.1016/S0140-6736(15)01020-X

[10] Lawn JE, Blencowe H, Waiswa P, Amouzou A, Mathers C, Hogan D, et al Stillbirths: rates, risk factors, and acceleration towards 2030. Lancet. 2016;387(10018):587–603. doi: 10.1016/S0140-6736(15)00837-5 . 26794078 10.1016/S0140-6736(15)00837-5

[11] Froen JF, Friberg IK, Lawn JE, Bhutta ZA, Pattinson RC, Allanson ER, et al Stillbirths: progress and unfinished business. Lancet. 2016;387(10018):574–86. doi: 10.1016/S0140-6736(15)00818-1 . 26794077 10.1016/S0140-6736(15)00818-1

[12] Stillbirths Registration Act 1994, (1994).

[13] O’Farrell IB, Manning E, Corcoran P, McKernan J, Meaney S, Drummond L, et al. Perinatal Mortality in Ireland Annual Report 2015. Cork: NPEC, 2017.

[14] Radestad I, Westerberg A, Ekholm A, Davidsson-Bremborg A, Erlandsson K. Evaluation of care after stillbirth in Sweden based on mothers’ gratitude. British Journal of Midwifery. 2011;19(10):646–52.

[15] Bennett SM, Litz BT, Maguen S, Ehrenreich JT. An Exploratory Study of the Psychological Impact and Clinical Care of Perinatal Loss. Journal of Loss & Trauma. 2008;13(6):485–510. doi: 10.1080/15325020802171268

[16] Flenady V, Wilson T. Support for mothers, fathers and families after perinatal death. Cochrane Database of Systematic Reviews [Internet]. 2008; (1). http://www.mrw.interscience.wiley.com/cochrane/clsysrev/articles/CD000452/frame.html. 10.1002/14651858.CD000452.pub2 18253978

[17] Schott J, Henley A. After a stillbirth—offering choices, creating memories. British Journal of Midwifery. 2009;17(12):798–801.

[18] Cacciatore J. The Unique Experiences of Women and Their Families After the Death of a Baby. Social Work in Health Care. 2010;49(2):134–48. doi: 10.1080/00981380903158078 . 20175019 10.1080/00981380903158078

[19] O’Connell O, Meaney S, O’Donoghue K. Caring for parents at the time of stillbirth: How can we do better?". Women & health. 2015. 10.1016/j.wombi.2016.01.003 26916147

[20] Mills TA, Ricklesford C, Cooke A, Heazell AE, Whitworth M, Lavender T. Parents’ experiences and expectations of care in pregnancy after stillbirth or neonatal death: a metasynthesis. BJOG. 2014;121(8):943–50. Epub 2014/03/04. doi: 10.1111/1471-0528.12656 . 24589119 10.1111/1471-0528.12656

[21] Ellis A, Chebsey C, Storey C, Bradley S, Jackson S, Flenady V, et al Systematic review to understand and improve care after stillbirth: a review of parents’ and healthcare professionals’ experiences. BMC Pregnancy Childbirth. 2016;16:16 doi: 10.1186/s12884-016-0806-2 . 26810220 10.1186/s12884-016-0806-2 PMC4727309

[22] Biggerstaff D, Thompson AR. Interpretative Phenomenological Analysis (IPA): A Qualitative Methodology of Choice in Healthcare Research. Qualitative Research in Psychology. 2008;5(3):214–24. doi: 10.1080/14780880802314304

[23] Smith JA, Flowers P, Larkin M. Interpretative Phenomonological Analysis: Theory, Method, Research. London: Sage; 2009.

[24] Nuzum DR, Meaney S, O’Donoghue K, Jackson M. Stillbirth and suffering in Ireland: a theological reflection from healthcare chaplaincy. Practical Theology. 2017;10(2). doi: 10.1080/1756073X.2017.1296062

[25] Nuzum D, Meaney S, O’Donoghue K. The Spiritual and Theological Challenges of Stillbirth for Bereaved Parents. Journal of Religion and Health. 2017:1–15. 28154999 10.1007/s10943-017-0365-5

[26] Peters MD, Lisy K, Riitano D, Jordan Z, Aromataris E. Providing meaningful care for families experiencing stillbirth: a meta-synthesis of qualitative evidence. Journal of perinatology: official journal of the California Perinatal Association. 2016;36(1):3–9. doi: 10.1038/jp.2015.97 . 26248132 10.1038/jp.2015.97

[27] Burden C, Bradley S, Storey C, Ellis A, Heazell AE, Downe S, et al From grief, guilt pain and stigma to hope and pride—a systematic review and meta-analysis of mixed-method research of the psychosocial impact of stillbirth. BMC pregnancy and childbirth. 2016;16:9 doi: 10.1186/s12884-016-0800-8 . 26785915 10.1186/s12884-016-0800-8 PMC4719709

[28] Eireann O. Protection of Life During Pregnancy Act 2013. In: Children DoHa, editor. Dublin: Government of Ireland; 2013.

[29] O’Connell O, Nuzum D, Meaney S, O’Donoghue K. Incompatible with Life but Not with Love: The Value of Prenatal Palliative Care in Cases of Lethal Abnormalities Diagnosed in the Prenatal Period. Journal of palliative care. 2014;30(3):219-.

[30] Meaney S, Gallagher S, Lutomski JE, O’Donoghue K. Parental decision making around perinatal autopsy: a qualitative investigation. Health Expect. 2014 doi: 10.1111/hex.12305 . 25376775 10.1111/hex.12305 PMC5810723

[31] Horey D, Flenady V, Heazell AE, Khong TY. Interventions for supporting parents’ decisions about autopsy after stillbirth. The Cochrane database of systematic reviews. 2013;2:CD009932 Epub 2013/03/02. doi: 10.1002/14651858.CD009932.pub2 . 23450611 10.1002/14651858.CD009932.pub2 PMC11625498

[32] Horey D, Flenady V, Conway L, McLeod E, Yee Khong T. Decision influences and aftermath: parents, stillbirth and autopsy. Health expectations: an international journal of public participation in health care and health policy. 2014;17(4):534–44. doi: 10.1111/j.1369-7625.2012.00782.x . 22708659 10.1111/j.1369-7625.2012.00782.x PMC5060741

[33] Heazell AEP, McLaughlin MJ, Schmidt EB, Cox P, Flenady V, Khong TY, et al A difficult conversation? the views and experiences of parents and professionals on the consent process for perinatal postmortem after stillbirth. BJOG: An International Journal of Obstetrics and Gynaecology. 2012;119(8):987–97. 22587524 10.1111/j.1471-0528.2012.03357.x

[34] Corcoran P, Manning E, O’Farrell IB, McKernan J, Meaney S, Drummond L, et al. National Perinatal Epidemiology Centre, Annual Report 2014. Cork: National Perinatal Epidemiology Centre, 2016.

[35] Nuzum D, Meaney S, O’Donoghue K. The public awareness of stillbirth: an Irish population study. BJOG. 2018;125(2):246–52. Epub 2017/10/30. doi: 10.1111/1471-0528.14939 . 28929637 10.1111/1471-0528.14939

[36] Warland J, O’Brien LM, Heazell AE, Mitchell EA, Consortium S. An international internet survey of the experiences of 1,714 mothers with a late stillbirth: the STARS cohort study. BMC Pregnancy Childbirth. 2015;15:172 Epub 2015/08/15. doi: 10.1186/s12884-015-0602-4 . 26276347 10.1186/s12884-015-0602-4 PMC4537542

[37] Downe S, Schmidt E, Kingdon C, Heazell AE. Bereaved parents’ experience of stillbirth in UK hospitals: a qualitative interview study. BMJ open. 2013;3(2). Epub 2013/02/19. doi: 10.1136/bmjopen-2012-002237 . 23418300 10.1136/bmjopen-2012-002237 PMC3586079

