# Supplementary material for: The impact of stillbirth on bereaved parents: A qualitative study
Source: PLoS One. 2018 Jan 24;13(1):e0191635. doi: 10.1371/journal.pone.0191635 (PMC5783401; doi:10.1371/journal.pone.0191635)
Supplement: S1 Appendix — (DOC) [file pone.0191635.s001.doc]

**S1 APPENDIX**

**Interview Schedule**

1: Introduction

2. Personal story:

Welcome and thank-you for participating.

Introductory information about the study and that participant understands the consent process and support available.

Participant asked to tell me their story from when they discovered they were pregnant through receiving news of stillbirth and the care they received and follow up care. (The following areas are for prompt)

What were your feelings when you discovered you were pregnant?

At what stage did you find out you were pregnant?

Do you have any other children? Ages, names, gender.

I wonder can you tell me about the time leading up to when you discovered that your baby {name} had died?

Did you have an inkling that something was wrong?

What were your feelings when you realised something was wrong?

Where were you told the news?

Who was with you?

How did you think the doctor/ midwife felt when they told you? (Were they comfortable? Were they sensitive?)

Can you remember the words he/she used?

What were your feelings towards your baby at that time?

Do you feel any sense of connection now towards your baby {name}?

Do you always feel this way or does it change at various times?

Did you have any sense of faith or belief before you heard that your baby {name} had died?

How would you describe it?

What were your feelings towards faith when you got the news?

Was there anything that you felt kept you going?

Did you feel that it was important to have any spiritual or religious ceremony for your baby? (blessing, naming, prayer service, funeral, cremation, burial etc)

What did you do?

Why?

Was it helpful?

Who facilitated this?

Is there anything you feel didn’t happen that you now wished had been offered to you?

Do you receive any mementos?

What were they?

Would you have liked your doctor to ask you about your spiritual concerns as part of their care of you? Is there anything else that we have not touched on that you feel is important in this area of care?
